# Hypocretin as a Hub for Arousal and Motivation

**DOI:** 10.3389/fneur.2018.00413

**Published:** 2018-06-06

**Authors:** Susan M. Tyree, Jeremy C. Borniger, Luis de Lecea

**Affiliations:** Department of Psychiatry and Behavioral Sciences, Stanford University, Stanford, CA, United States

**Keywords:** hypocretin, lateral hypothalamus, arousal, motivation, reward

## Abstract

The lateral hypothalamus is comprised of a heterogeneous mix of neurons that serve to integrate and regulate sleep, feeding, stress, energy balance, reward, and motivated behavior. Within these populations, the hypocretin/orexin neurons are among the most well studied. Here, we provide an overview on how these neurons act as a central hub integrating sensory and physiological information to tune arousal and motivated behavior accordingly. We give special attention to their role in sleep-wake states and conditions of hyper-arousal, as is the case with stress-induced anxiety. We further discuss their roles in feeding, drug-seeking, and sexual behavior, which are all dependent on the motivational state of the animal. We further emphasize the application of powerful techniques, such as optogenetics, chemogenetics, and fiber photometry, to delineate the role these neurons play in lateral hypothalamic functions.

## Introduction

Through decades of neuroanatomical and behavioral work, the lateral hypothalamic area (LHA) has been delineated as a key region for the integration and regulation of sleep, feeding, stress, energy balance, reward, and motivated behavior. Further in-depth analyses of molecular, cellular, and circuit-level functions of the LHA have made progress in gaining a holistic understanding of how the LHA integrates sensory information to influence arousal and behavior. The lateral hypothalamic cell population that has been the most intensely studied thus far is a group of neurons expressing the neurotransmitters hypocretin-1 and -2 (also known as orexin-A and -B). Cells expressing these peptides span the lateral and perifornical/medial hypothalamus (throughout the tuberal hypothalamus). These cells were identified by two groups at essentially the same time. The first approach used directional tag PCR subtraction, a technique adapted to enrich mRNAs that were specifically expressed in the hypothalamus (1). They observed a hypothalamic-specific mRNA (prepro-hypocretin), which encoded two putative protein products (Hcrt-1 and -2). This group named the newly identified neurotransmitters hypocretins after the name was voted on at an annual Society for Neuroscience meeting, the rationale being their restricted hypothalamic localization and their similarities to the gut peptide secretin. They found that these peptides were present in synaptic vesicles, and one of these peptides could stimulate hypothalamic neurons *in vitro*, suggesting it acted as a neurotransmitter. Another group identified the same peptides via ligand screening of multiple orphan G-protein coupled receptors (GPCRs) using a cell-based reporter system, and, because they promoted feeding, named them “orexins” (2). Over the last 20 years, it has become apparent that the essential purpose of the hypocretins/orexins seems to be for wake maintenance (hypocretin deficiency results in the sleep disorder narcolepsy, discussed below) and not feeding. Therefore, we refer to them as the hypocretins throughout this manuscript.

**Figure 1:**
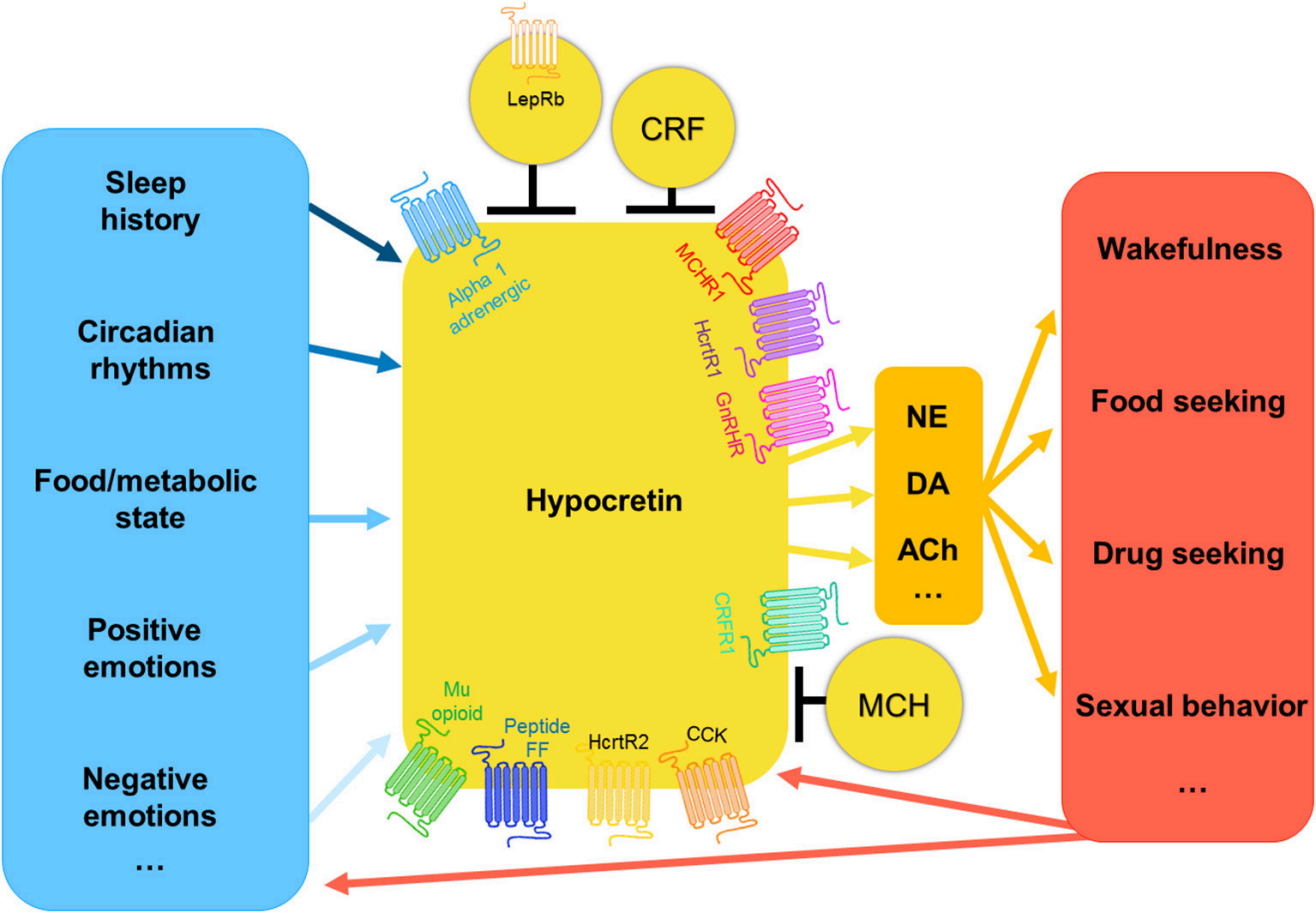
The hypocretin system: inputs and outputs. Hypocretin neurons receive information regarding nutritional state, emotional state, and environmental cues regarding temperature, time of day, and recent sleep history, the weight that these inputs have is very likely variable and interrelated. Hypocretin-expressing neurons are very heterogeneous, they are known to co-express numerous other genes. Additionally, hypocretin-expressing neurons are modulated by the activity of surrounding neuron populations present in the lateral hypothalamus, including melanin-concentrating hormone, leptin receptor-expressing neurons, and corticotropin releasing factor neurons. Hypocretin neurons project to many neuron populations throughout the brain, including norepinephrine, dopaminergic, and cholinergic neurons. It is via these pathways that the hypocretin system modulates the wide range of outputs related to arousal and motivated behavior. In turn, the consequences of these behaviors go on to inform the hypocretin system as to the newly updated status of the animal, and thus determine the next hypocretin-driven behavioral output.

It is unsurprising that hypocretin neurons are fundamental stabilizers of wakefulness as well as powerful modulators of motivated behavior. The hypocretins were found to be expressed exclusively in an LHA population of glutamatergic cells, and project to most nuclei that regulate sleep/wake behavior (3). Hypocretin (Hcrt) neurons are extremely heterogeneous (Figure 1), this heterogeneity has recently been explored using single cell sequencing to show the large number of genes that are co-expressed within Hcrt cells (4). Hcrt neurons are transcriptionally and electrophysiologically distinct from co-mingled neurons expressing melanin concentrating hormone [MCH; (5, 6)], and they are most strongly active during active wakefulness, decrease activity during quiet wakefulness, and are silent during NREM and REM sleep (7, 8). This is essentially the opposite firing pattern displayed by neurons expressing MCH, which innervate many of the same targets as Hcrt neurons (9, 10). Indeed, evidence suggests inhibitory signaling from Hcrt to MCH neurons prevents simultaneous activation of both neural populations (11). Hcrt and MCH neural populations can be further discriminated based on their expression of key transcription factors [Lhx9; 100% of Hcrt neurons, 3.4% of MCH neurons; and Nkx2.1: 60.1% of MCH neurons and 0% of Hcrt neurons; (4)]. Afferent inputs to Hcrt neurons were mapped using a combination of tract tracing methods, uncovering major projections from the lateral septal nucleus, bed nucleus of the stria terminalis, preoptic area, multiple hypothalamic nuclei, substantia nigra, and ventral tegmental area (VTA), as well as the dorsal raphe (12). Genetic tracing studies revealed cell-type specific inputs arriving from cholinergic neurons in the laterodorsal tegmentum, preoptic GABAergic neurons, as well as 5-HT neurons in the median/paramedian raphe, suggesting a major role for these neurons in functions ranging from neuroendocrine control to arousal and metabolic processes (13).

Highlighting its essential role in vigilance state stability is the conservation of the Hcrt peptides throughout the phylogenetic tree. For example, hypothalamic Hcrt expression can be detected in the zebrafish embryo 22 h post-fertilization (14), and Hcrt overexpression causes an insomnia-like phenotype in adult zebrafish (15). Further studies have demonstrated that Hcrt neurons send widespread projections throughout the zebrafish brain that participate in the consolidation of wakefulness, as they do in mammals (16, 17). Indeed, genetic destruction of Hcrt neurons fragments vigilance states in zebrafish (18, 19). In cavefish, Hcrt signaling seems to play a role in the evolution of sleep loss. Hcrt is expressed 3-fold more in cave vs. surface fish, and these fish sleep much less than their surface-dwelling counterparts. Indeed, manipulations that promote sleep inhibit Hcrt expression in cave, but not closely related surface fish (20). Hcrts are also expressed in the avian brain, where they are localized to a single population of neurons spanning the paraventricular and lateral hypothalamus (21, 22).

## Arousal transitions

During World War I, Viennese neurologist Baron Constantin von Economo meticulously analyzed and documented the symptoms of a novel condition he termed “encephalitis lethargica” (23). The causative agent for the condition (presumed viral or autoimmune) remains unknown, but patients showed prolonged states of sleep (>20 h/day) and they could only be awakened with strong and sufficient stimulation (24). He noted that many of these patients had significant damage to the posterior hypothalamus (containing the LHA) and rostral midbrain, while patients showing the opposite problem (prolonged insomnia) had damage to the basal forebrain and preoptic area (25). Based on these observations, he predicted that neurons in the anterior hypothalamus promote sleep while those in the posterior region promote wakefulness. During the following years, his predictions were largely substantiated in a number of species including monkeys (26), rats (27), and cats (28). With the application of the electroencephalogram (EEG; discussed below) to sleep/wake analyses (29), as well as significant advancements in molecular biology, different cell groups within the hypothalamus have been implicated in controlling arousal.

In all mammals (and many non-mammalian vertebrates), sleep stages and structure can be objectively determined via comparative analysis of the electroencephalogram (EEG) and electromyogram (EMG) signals. The EEG reflects large scale changes in electrical activity in the cerebral cortex, as the firing rate of cortical neurons steadily declines during non-rapid eye movement (NREM) sleep in comparison to rapid eye movement (REM) sleep and wakefulness (30–32). Aggregate firing rates of cortical ensembles (reflected in the EEG) can be parsed into conventional bandwidths at approximate frequencies: alpha (9–12 Hz), beta (12–30 Hz), delta (i.e., slow-waves; 0.5–4 Hz), low (30–60 Hz), and high (60–100 Hz) gamma, and theta (5–9 Hz). Synchronization (reflected in high-voltage, low frequency oscillations) of the cortical EEG during NREM sleep depends on a corticothalamocortical loop, which is modulated by local oscillators and is distally controlled by subcortical systems [including those located in the LHA; (33, 34)]. During NREM sleep, delta waves dominate the EEG, and EMG activity (postural tone) is low or absent. During wakefulness, EMG activity is high reflecting muscle activity, and the EEG shows task dependent spectral properties, with low theta frequencies building over time indicating growing sleep propensity (35). REM sleep, also known as paradoxical sleep, is characterized by a wake-like (high theta) EEG spectra composition, but (paradoxically) low or absent EMG activity.

The sleep disorder narcolepsy [characterized by daytime sleepiness, aberrant transitions from wakefulness into REM sleep, and spontaneous loss of muscle tone, referred to as cataplexy; (36)] highlights the essential role of Hcrt in arousal regulation. Loss of Hcrt or Hcrt receptors causes a narcolepsy-like phenotype in mice, including cataplexy and altered sleep/wake cycles (37, 38). Additional canine data identified a genetic defect in Hcrt receptor 2 (HcrtR2) linked to the development of narcolepsy in dogs (36), and several studies have confirmed that degenerative loss of Hcrt neurons is responsible for human narcolepsy (39–41). Degenerative cell loss, rather than reduced Hcrt gene expression is the likely cause because other markers that co-localize within Hcrt neurons are reduced in narcoleptic patients as well (42). Hcrt is a primary stabilizer of wakefulness, as narcoleptics or transgenic mouse models of the disorder display normal amounts of sleep, but with inappropriate transitions between each vigilance state (43, 44). This suggests that Hcrt neurons serve a surprisingly non-redundant role in the regulation of wakefulness, as their loss creates such a marked effect on the stability of arousal.

Initial microdialysis (45) and cFos immunoreactivity (46) studies demonstrated a circadian modulation of Hcrt neural activity, where Fos expression within Hcrt neurons peaked during the active (dark) phase in rodents. Subsequent juxtacellular recordings in head-fixed or freely moving animals demonstrated that Hcrt activity is largely phasic and firing rates peak prior to sleep-to-wake transitions by approximately 10–20 s (7, 8). Building on these studies, the development of optogenetic technologies for manipulating brain circuits has contributed significantly to the investigation of arousal circuits (47). Indeed, the first primary functional evidence for Hcrt's role in wakefulness was presented in 2007, where the first *in vivo* use of optogenetics revealed that light-evoked stimulation of Hcrt neurons powerfully promoted wakefulness in mice, an effect dependent on Hcrt (and not other co-released neurotransmitters) signaling (48). The effect was frequency-dependent, as stimulations below 5 Hz failed to alter the probability of sleep-to-wake transitions. Further studies demonstrated that long-term optogenetic silencing of Hcrt neurons promotes slow-wave sleep in mice, although only during the light (inactive) phase (49). These optogenetic findings were recapitulated via chemogenetic experiments where designer receptors exclusively activated by designer drugs (DREADDs) were expressed in Hcrt neurons. Engaging excitatory Gq signaling in Hcrt neurons increased cFos immunoreactivity and promoted wakefulness while inhibitory Gi signaling promoted sleep (50). More recent work has investigated exactly which components of wakefulness for which Hcrt is necessary. For example, Vassali & Franken observed that Hcrt-deficient mice had marked reductions in theta-dominated wakefulness during baseline recordings, an oscillatory pattern associated with goal-driven and exploratory behavior (51). Upon forced wakefulness however, normal amounts of theta-dominated wakefulness persisted. These data suggest that Hcrt is necessary for spontaneous, but not enforced wakefulness, which may rely on other neuromodulators. This avenue of research has led to the development of novel drugs for insomnia. As insomnia is thought to be the result of overactive wake-promoting neural systems (52), Hcrt receptor antagonists have been developed as a targeted therapeutic approach. Suvorexant (Belsomra) is the first dual Hcrt receptor antagonist approved for use by the USDA (in 2014) and is now prescribed for insomnia throughout the USA and Japan.

### Efferents involved in cortical arousal

How do these cells in the subcortical hypothalamus promote activation and desynchronization of the cortex? One possibility is that diffuse projections from Hcrt neurons generally promote arousal across the brain. Alternatively, specific projections may be essential for their arousal-promoting properties. Direct actions of the Hcrts on arousal-promoting nuclei has been demonstrated for locus coeruleus (LC) noradrenergic neurons, 5-HT neurons in the dorsal raphe, dopaminergic (DA) neurons within the VTA, histamine neurons in the tuberomammillary nucleus, and cholinergic neurons in the brainstem and basal forebrain (53). Below, we discuss the varying degrees of evidence supporting the role of Hcrt in regulating the arousal-promoting effects of each of these areas.

#### Locus coeruleus (LC)

The noradrenergic LC has long been recognized to promote wakefulness and arousal (54, 55), and it receives the densest input from Hcrt neurons (3). Direct administration of Hcrt to the LC depolarizes neurons in this region and increases LC firing rates, which is associated with arousal (56–59). Further studies involving Hcrt stimulation with optogenetic silencing of the LC revealed that it is a critical hub for relaying the signal from the LHA to promote wakefulness (57, 60), as activation of Hcrt neurons with simultaneous inhibition of LC prevented sleep-to-wake transitions. Additionally, increasing the excitability of LC neurons using step function opsins (SFOs) was shown to facilitate Hcrt-mediated wakefulness.

#### Basal forebrain (BF)

The basal forebrain is a heterogenous structure composed of cholinergic and non-cholinergic (mostly GABAergic) neurons with differential effects on arousal, spanning the substantia innominata, the vertical and horizontal limbs of the diagonal band, the extended amygdala, ventral pallidum, and the medial septum (61). These areas receive moderate to heavy input from Hcrt neurons (3). The arousal promoting effects of basal forebrain stimulation has been extensively characterized using pharmacological, electrical, and chemical ablation methods (62–65). Using cell-type specific optogenetic tools, stimulation of cholinergic neurons within the basal forebrain was shown to be sufficient for cortical activation and transitions out of NREM sleep (66, 67). Additional targeting of BF GABAergic neurons demonstrated their role in cortical activation and wakefulness (68). BF cholinergic neurons were demonstrated to fire predominantly during cortical activation rather than slow-wave activity and are activated by Hcrt (69). Injection of Hcrt into the basal forebrain stimulates cortical activation and wakefulness (70). Hcrt-1 is released in the BF during wakefulness (71), where it has differential actions on cholinergic and non-cholinergic neurons (72, 73), leading to cortical release of acetylcholine and arousal [for review, see (74)].

#### Tuberomammillary nucleus (TMN)

The TMN contains histaminergic neurons that are silent during sleep and begin to fire after wake onset, where histamine release promotes arousal (75–77). Acute silencing of TMN histamine neurons inhibits wakefulness and rapidly induces NREM sleep (78). This area receives moderate to dense input from Hcrt neurons (3). Hcrt was shown to directly activate histaminergic neurons in the TMN (79, 80), and further modulate GABAergic inputs to this region (81). Additional optogenetic studies provided evidence that Hcrt neurons are capable of fast glutamatergic control of histaminergic neurons in the TMN (82). However, Hcrt inputs to TMN histaminergic neurons, in contrast to the LC, do not seem to be important for regulating sleep-to-wake transitions, as stimulation of Hcrt neurons is sufficient to promote awakening in mice lacking histidine decarboxylase [the rate limiting enzyme in histamine synthesis; (83)]. Further optogenetic manipulation of Hcrt fibers in the TMN is necessary to effectively delineate the role this circuit plays in sleep/wake regulation.

#### Dorsal raphe nucleus (DRN)

The DRN has long been implicated in arousal, showing vigilance state-specific changes in firing rates (84). Composed of primarily serotonergic (5-HT) neurons, they are excited by multiple arousal-related peptides, including Hcrt (85–88). Hcrt neurons receive reciprocal input from 5-HT neurons, which directly inhibit them via the 5HT1A receptor, and indirectly via enhancement of GABAergic inputs (89). Direct stimulation of 5-HT-expressing DRN neurons demonstrated that ChR2-mediated excitation of these cells caused an immediate transition from NREM sleep to wakefulness (90), and an additional population of DA neurons in this region was recently implicated in arousal regulation (91). Similar to the TMN, direct (i.e., cell-type and circuit specific) investigation of Hcrt's actions on DRN 5-HT or DA neurons in arousal regulation has yet to be completed.

#### Laterodorsal tegmental nucleus (LDT)

The laterodorsal tegmental nucleus (LDT) is a key pontine site in the regulation of wakefulness (92), primarily composed of cholinergic and non-cholinergic neurons (93). Optogenetic activation of LDT cholinergic neurons increases REM sleep episodes (but not duration) when stimulations were done during NREM sleep (94). Hcrt-1 injections into the LDT promote wakefulness in cats, suggesting that an arousal circuit from Hcrt to the LDT exists (95). Further studies revealed that Hcrt acts on LDT neurons at both pre- and post-synaptic sites. Pre-synaptically, Hcrt increases the amplitude and frequency of spontaneous excitatory post-synaptic currents (EPSCs) via triggering action potentials and enhancing synaptic transmission in glutamatergic nerve terminals (96). Post-synaptically, Hcrt promotes an inward current in tandem with enhanced membrane current noise in both cholinergic and non-cholinergic LDT neurons (96). Similar to other areas studied, application of optogenetic tools to Hcrt projections to LDT neurons has not been described, which would directly integrate the LDT into the Hcrt arousal network.

#### Ventral tegmental area (VTA)

The VTA is composed primarily of DA and GABAergic neurons and has long been recognized as a node regulating arousal and motivation (97, 98). Early electrophysiological studies suggested that VTA and substantia nigra pars compacta neurons do not show behavioral-state dependent alterations in firing rate (97–101). However, subsequent studies challenged this view, where both DA and GABA neurons within the VTA show vigilance-state dependent changes in activity (102, 103). It was only recently that causal evidence was presented demonstrating the role of VTA-DA neurons in vigilance state switching, where DA neurons strongly promote wakefulness and are primarily active during wakefulness and REM sleep (104–106). Hcrt neurons send moderate to dense projections to the VTA, where they activate both DA and non-DA neurons (107, 108). Additionally, Hcrt infusion into the lateral ventricles or directly into the VTA increases DA efflux in the pre-frontal cortex, suggesting that activation of VTA-DA neurons contributes to the vigilance-state modulatory properties of Hcrt (109). Whether a circuit from Hcrt to VTA-DA or GABAergic neurons causally promotes vigilance state switching remains to be determined.

#### Distributed and indirect actions

Hcrt peptides can also have direct effects on cortical neurons. These neurons innervate deep layers of the cerebral cortex (3), and more recent studies suggest that layer VIb cortical neurons directly respond to Hcrt peptides (110, 111). nNOS/NK1R expressing cortical interneurons [which play a putative role in sleep homeostasis (112)] express HcrtR1 mRNA (113). A subpopulation of these interneurons is responsive to Hcrt-1, but lack of Hcrt input to these cells does not influence their ability to detect sleep pressure (114). Indeed, further research is required to gain an understanding of direct Hcrt effects on cortical neural populations.

Recently, brain interstitial ion concentrations have been shown to directly influence sleep and wakefulness, providing an indirect pathway by which Hcrt-ergic signaling may influence arousal (115). Neuromodulators (e.g., dopamine, norepinephrine, hypocretin) all alter membrane potentials to influence spiking properties and intracellular signaling components. In doing so, network-wide changes in ionic concentrations must occur, which in turn may further influence vigilance states to ensure concerted brain activity. Nedergaard and colleagues discovered that independent of synaptic activity, altering the concentrations of extracellular K^+^, Ca^2+^, Mg^2+^, and H^+^ was able to cause a reversible switch between sleep and wakefulness. This suggests that downstream of neuromodulator actions (e.g., Hcrt), the build-up of certain ionic concentrations in the brain may independently drive sleep pressure and alter the wakefulness threshold.

### Regulation of hypocretin neural activity

Hcrt neurons respond to a wide variety of neurotransmitters and hormones (116), including NE, 5-HT, NPY, CCK, ghrelin, acetylcholine, and glutamate (117). These factors contribute to the ability of Hcrt neurons to respond to arousal and metabolic signals to adjust vigilance states accordingly. Hcrt neurons are further activated by corticotropin-releasing factor [CRF; (118)] and thyrotropin-releasing hormone [TRH; (119)], while they are generally inhibited by GABA (120) and adenosine (121). Noradrenergic, serotonergic, and DA inputs negatively regulate Hcrt activity, while histaminergic inputs have little effect (122–125). Other metabolic factors (e.g., ghrelin, leptin, glucose) that regulate their activity are discussed in section Feeding Behaviors and Metabolism.

Do Hcrt receptors (HcrtR1 and HcrtR2) share redundant functions in the regulation of arousal? HcrtR1 binds Hcrt-1 preferentially over Hcrt-2, while HcrtR2 binds both peptides at high affinity (2). These receptors show differential expression patterns across the mammalian brain, with HcrtR1 most abundant in the ventromedial hypothalamus, tenia tecta, hippocampus, dorsal raphe, and LC. HcrtR2 mRNA was localized to the paraventricular hypothalamus, cerebral cortex, nucleus accumbens, paraventricular thalamus, and anterior pretectal nucleus (126). HcrtR2-deficient mice show a phenotype similar to narcolepsy, including fragmented wakefulness, while those deficient in HcrtR1 show only mild sleep alterations (127, 128). Double receptor-knockout mice, however, show an even stronger phenotype than the HcrtR2 knockouts alone, including cataplexy and REM sleep intrusion into wakefulness, suggesting some redundancy in receptor function (37, 129). However, an fMRI study demonstrated that antagonism of HcrtR2 but not HcrtR1 increased REM and NREM sleep time, suggesting distinct roles of the receptors (130). The picture is not so clear though, as HcrtR1 blockade can influence the effects of HcrtR2 antagonism, suggesting complex interactions among Hcrt receptors (131).

#### Circadian rhythmicity and hypocretin

How do Hcrt neurons integrate time-of-day information to appropriately promote arousal and motivated behavior? The circuitry discussed above largely controls vigilance state switching on short to medium (1–30 s) timescales, so there must be some intervening population of cells that conveys longer term (i.e., hours to days) information to Hcrt neurons to regulate their activity. No direct synaptic connections between the master clock located in the suprachiasmatic nuclei (SCN) and Hcrt neurons has been demonstrated (132). However, in dark pulse experiments, where a 6 h pulse is given during the subjective day (a stimulus that promotes arousal and phase advances behavioral rhythms in mice), a pulse of dark is sufficient to release Hcrt neurons from SCN-mediated inhibition (133), providing evidence for light-dependent suppression of Hcrt signaling via the SCN. Because Hcrt neurons receive synaptic input from the dorsomedial and paraventricular hypothalamus (13, 134), which are primary outputs of the SCN (135), polysynaptic communication between the core clock and lateral hypothalamic Hcrt neurons is a likely mediator of this effect.

Hcrt neurons themselves seem to show a circadian rhythm in structural plasticity. In zebrafish, two-photon imaging of Hcrt axons revealed day/night differences in synapse numbers, which was driven by changes in *nptx2b*, a gene implicated in AMPA receptor clustering (136). Overexpression of this gene in Hcrt neurons further rendered fish resistant to the sleep-promoting effects of melatonin, providing evidence for a behavioral effect of circadian structural plasticity within the Hcrt system. Reciprocally, Hcrt neurons send inputs to the SCN to modulate clock function. SCN neurons express Hcrt receptors (137), suggesting there may be interplay between the two neural populations to coordinate phase coherent output of the SCN coinciding with behavioral arousal. This poses a problem, though, as Hcrt signaling is generally excitable, but SCN neural activity is low during the circadian night when Hcrt activity is high (in nocturnal rodents). A few years ago, it was discovered that unlike other connections, projections from Hcrt to the SCN were suppressive, and the mechanisms mediating this effect varied in a circadian fashion (138). By examining calcium activity in SCN neurons, both spontaneous and in response to Hcrt administration, it was demonstrated that a subset showed reduced (69%) or increased (31% cytosolic Ca^2+^ levels in response to Hcrt during the day, and 97% reduced Ca^2+^ activity in response to Hcrt at night. This response depended on HcrtR1 activation, as treatment with the HcrtR1 antagonist (SB334867) prevented this response but did not interfere with NMDA-induced Ca^2+^ increase. The suppressive effects of Hcrt seems to further depend on GABAergic signaling within the SCN, as the inhibitory effect is blocked when Hcrt is applied in the presence of gabazine and CGP55845 (antagonists of GABA_A_ and GABA_B_ receptors). These observations provide evidence for a reciprocal relationship between the SCN and Hcrt neurons in the lateral hypothalamus, demonstrating suppressive and phase-modulatory actions (through Ca^2+^) of Hcrt on the circadian clock.

### Hyper arousal in response to stressors

The term arousal covers more than mere wakefulness, indeed, it can be considered an umbrella term for multiple waking states, such as restful waking, active waking, and stressed states. Due to the well-studied role for Hcrt initiating arousal from sleep, researchers have also investigated the possibility of a role for Hcrt in the transition from arousal into hyper-arousal associated with stressed states. Supporting evidence for this role has been found in patients suffering from panic symptoms who have elevated levels of Hcrt in their cerebrospinal fluid in comparison to control subjects without substantial panic symptoms (139). Additionally, in animal studies it has been shown that multiple different stressors can trigger changes in Hcrt levels, including restraint stress, cold stress (140), and chronic social stress (141). In line with this finding, studies observing neural activation of Hcrt neurons have shown that stressors such as the forced swim test or indeed, direct infusion of corticotropin-releasing factor result in increased Hcrt activity (118, 142), showing a direct effect of physiological stress responses on Hcrt activity.

In order to further investigate the role of Hcrt in stress researchers have also investigated how manipulation of Hcrt function affects an animal's response to stress. Central administration of Hcrt-1 results in an increase in anxious behaviors as measured in multiple standard laboratory tests of anxiety such as the elevated plus maze, the light-dark box exploration test (143), the open-field test, and novel object exploration (144). Pharmacological administration of Hcrt has also been shown to stimulate hypothalamic-pituitary-adrenal (HPA) axis, resulting in increased concentrations of adrenocorticotropic hormone and corticosterone in plasma (118, 142, 145, 146). In line with these findings, impairing Hcrt function appears to reduce behavioral markers of anxiety and stress responses. Hcrt-deficient mice show a reduction in the exhibition of stress responses, such as changes in blood pressure, heart rate, respiratory changes (147, 148), as well as behavioral responses to resident intruders (147). These results suggest an important role for Hcrt in normal physiological and behavioral stress responses and confirm that these responses are impaired when animals do not have a fully functioning Hcrt system.

Following the development of optogenetic technologies and chemogenetics researchers have been able to genetically target specific neurons populations. Bonnavion et al. (149) used optogenetics to further investigate the role of Hcrt as a potential trigger for HPA axis activation, and showed that optogenetic stimulation of Hcrt neurons is sufficient to induce HPA axis activation which resulted in physiological stress responses including significantly elevated plasma corticosterone secretion and elevated heart rate, suggesting that increased Hcrt activity is a physiologically significant stressor. Bonnavion et al. also observed that this Hcrt-induced stress response could be mediated by nutritional status of the mouse, with food restricted mice showing a significantly higher Hcrt-induced stress response compared to *ad libitum*-fed controls. Further investigation of interactions between Hcrt, feeding, and stress showed that satiety hormone leptin, which, itself has been shown to be behaviorally anxiolytic (150, 151), modulates HPA axis activation generally, as well as Hcrt-induced HPA axis activation specifically (149). Food interactions with Hcrt activity will be discussed in more depth in section Feeding Behaviors and Metabolism. The development of chemogenetic technologies has also allowed the investigation of the role of Hcrt in resiliency to repeated social stress (152). After discovering that socially resilient rats (as tested in a social defeat assay) showed significantly lower levels of prepro-hypocretin mRNA, (152) went on to chemogenetically inhibit Hcrt neurons during the same social defeat paradigm, and found that reducing Hcrt activity during the social defeat paradigm caused rats to express fewer depressive-like behaviors and increased social interaction, both markers of a socially resilient phenotype. Taken together, these studies have used genetically targeted methods to show that inhibiting Hcrt can reduce stress-related behaviors, and that activation of Hcrt neurons can produce physiological stress responses, suggesting an important role for Hcrt in driving stress responses.

#### Hypocretin and immune stress

The Hcrt system is also influenced by immune stress. Indeed, it has been suggested that narcolepsy may have an autoimmune origin [for review, see (153)]. Specific genotypes in the antigen presentation complex HLA-DQB1 (HLA DQB1^*^06:02) are strongly associated with narcolepsy (154). Five epitopes from the Hcrt precursors are predicted to bind HLA DQB1^*^06:02, however, a recent study failed to find evidence of autoreactive CD4^+^ T-cells targeting Hcrt precursors (155), and a prior study claiming CD4^+^ T-cell autoimmunity to Hcrt and cross-reactivity to an epitope present in the 2009 H1N1 influenza virus was retracted when the authors failed to replicate their own findings (156). During the H1N1 (aka “Swine flu”) pandemic in 2009, cases of childhood narcolepsy rose dramatically in relation to administration of the vaccine (157). This effect was linked to the adjuvant (AS03, a squalene-based adjuvant from GlaxoSmithKline) co-administered with the vaccine (158, 159), although one study did not find an association (160). Additionally, in a mouse model of H1N1 infection, the virus was shown to enter sleep-wake regulatory regions and promote a narcolepsy-like phenotype (161). Despite this and other associative evidence, neither the essential targets nor a specific molecular mechanism have been identified linking narcolepsy to autoimmunity.

Less well studied is how Hcrt neurons sense the inflammatory milieu to regulate arousal/motivation. Peripheral lipopolysaccharide (LPS) challenge reduces cFos expression in Hcrt neurons as well as Hcrt concentrations in cerebrospinal fluid in rats (162). This is associated with reduced physical activity characteristic of “lethargy.” This, along with the finding that administration (intracerebroventricular) of Hcrt-1 counteracts LPS-induced reductions in locomotor activity, suggests that these neurons sense the inflammatory environment. However, this paper (162) found little evidence for direct targeting of Hcrt neurons by inflammatory cytokines. Instead, LPS seemed to activate upstream neurotensin neurons, which send inhibitory (GABAergic) projections to Hcrt cells to regulate their activity. Another study found that cytotoxic chemotherapy-induced fatigue was associated with reduced Hcrt cFos expression in conjunction with neuroinflammation, and treatment with Hcrt-1 rescued chemotherapy-induced reductions in locomotor activity (163). Inhibition of Hcrt activity by inflammatory mediators seems to be adaptive, as mice lacking Hcrt neurons (ataxin-3 transgenic) show enhanced recovery (NREM sleep) following LPS challenge (164). However, a more recent study demonstrated that peripheral administration of Hcrt-1 improves survival in mice given a lethal dose of LPS (165). An early study suggested that Hcrt-1, but not Hcrt-2, crossed the blood-brain-barrier [BBB; (166)], however, subsequent research has suggested that Hcrt-1 cannot normally cross the BBB except in high-dose administrations (167). Alternatively, systemic inflammation in response to LPS can disrupt BBB function and permit entry of this peptide into the brain. ICV-infusion of Hcrt-1 peptide recapitulates the enhanced survival phenotype shown in mice that received peripheral Hcrt-1. Because Hcrt neurons send projections to sympathetic output nuclei (3), and noradrenergic signaling potently alters peripheral immune function (168), a feedback loop between central and peripheral immune factors may be modulated by Hcrt to gauge arousal/motivation based on inflammatory status.

## Motivation

There are a number of VTA-projecting neuron types in the lateral hypothalamus that are considered to play a role in reward processing. Excitotoxic lesions of the LHA produce similar pathologies to that of dopamine depletion, including aphagia (169, 170), suggesting that LHA input to the VTA is critical for modulating motivated behavior. Indeed, both VTA DA and GABAergic neurons receive robust input from diverse LHA cell groups (171, 172). With cell-type specific tools (e.g., Cre-dependent viral vectors carrying optogenetic payloads), the role specific LHA cell types play in VTA-elicited behavior is becoming clearer. For example, by using real time place preference (RTPP) and intracranial self-stimulation (ICSS) experiments, Tye and colleagues were able to demonstrate that LHA-GABAergic and LHA-glutamatergic projections to the VTA promote approach and avoidance behaviors, respectively (173). Furthermore, inhibiting the LHA-GABA projections to the VTA while mice were in a motivated state (i.e., food restricted) reduced feeding time suggesting a reduction in the motivation to eat. These responses were observed to be related to elicited dopamine release in the downstream nucleus accumbens (NAc), as LHA-GABA stimulation enhanced while LHA-glutamatergic stimulation suppressed dopamine release in this brain region [for review, see Tyree et al. (174)].

### Feeding behaviors and metabolism

Hcrt is known to play a role in feeding behaviors. Indeed, studies have shown that Hcrt administration results in increased food intake [Hcrt-1 and Hcrt-2 i.c.v., (2); Hcrt-1 into NAc shell, (175)] and can increase motivation for food reward in a progressive ratio schedule task (176, 177). Furthermore, temporary antagonism of HcrtR1 (via SB334867) reduces food intake (178), reduces both motivation for (176, 179, 180)and consumption of (177, 180) food reward in a progressive ratio schedule task, and is sufficient to attenuate the effect of Hcrt-1-induced increase in feeding behaviors (181). Taken together, these findings suggest that Hcrt plays a role in driving food-intake and motivating food-seeking behaviors. However, there is some evidence that the nutritional status of the subject, and the type of food reward being presented can both mediate the effectiveness of Hcrt in driving these behaviors, interestingly, a study showed that blocking HcrtR1 significantly reduced the motivation to work for the delivery of cocaine as well as a highly palatable high fat diet, but had no effect on the motivation to work for regular chow food pellets (180).

It is clear that temporary changes in Hcrt activity can modulate feeding and food seeking behaviors, however more chronic manipulation of Hcrt suggests a more complex role for Hcrt in modulating food intake. While it is apparent that Hcrt knockout mice do show a reduction in the intake of food (38, 182) it appears that they still go on to develop obesity later on Hara et al. (38), suggesting that it may not only be food-seeking and food-consumption that is being driven by Hcrt activity, but additional processes underlying food-metabolism as well. This would be in line with previous findings that both gastric acid secretion (183) and metabolic rate (184) are stimulated following Hcrt administration. Additionally, in two obese mouse models (*ob/ob* and *db/db*) both strains of mice showed a significant downregulation of the prepro-hypocretin gene (185). These findings could signify that Hcrt may not only be important for driving metabolic processes important for weight maintenance, such as metabolic rate, but that their activity is also affected when metabolic state is altered, for instance in mouse models of obesity.

Hcrt neurons are specially situated to receive and integrate information on peripheral signals such as dietary content, metabolic status, and immune challenge due to their location in the LHA and there is evidence that altering food-intake also alters Hcrt activity. Hcrt neurons sense energy balance by monitoring glucose concentrations in the extracellular space through the use of a specialized 'tandem-pore' potassium (K^+^) channel (K_2P_) (186). Forty-eight hours of fasting can result in the upregulation of hypothalamic prepro-hypocretin mRNA (2, 187), this is consistent with the finding that insulin-induced hypoglycemia also increases the amount of prepro-hypocretin observed in the LHA (188). Conversely, glucodeprivation (induced by i.p. administration of 2-deoxy-D-glucose) decreased prepro-hypocretin (189), or had no effects (187, 190). The evidence of downregulation of prepro-hypocretin in obese mouse models (185) suggests that Hcrt activity is also being driven by dietary signals that are impaired in obesity. Both *ob/ob* and *db/db* mouse models are based on a deficiency of, or reduced responsiveness to leptin (191–195) a hormone that is released in response to dietary fat intake (196) and is commonly referred to as a satiety hormone. Hcrt neurons express receptors for leptin (197) and are inhibited by administration of leptin (198). Local GABAergic neurons that express the leptin receptor (LepRb) are additional negative regulators of Hcrt activity (149). Recent experiments using fiber photometry to monitor Hcrt neural activity during feeding behavior revealed that these cells rapidly reduce activity upon eating [even when the 'food' eaten is calorie free; (134)]. These findings suggest that the release of dietary hormones following food intake inhibits Hcrt neuron activation, which has been shown to drive food intake and food seeking. Then, the satiety response resulting from leptin and insulin release following food intake is mediated via the inhibitory effect these hormones have on the Hcrt system.

Hcrt activity is closely linked to not only leptin and insulin, both of which are associated with reducing food intake, but also to the hunger-associated hormone ghrelin which promotes feeding (199–205). Hcrt levels are directly modulated by these food-related hormones—as leptin and glucose levels increase and ghrelin levels decrease (as would occur following food consumption) Hcrt activity increases (117, 186, 206, 207). Additionally, ghrelin-induced feeding can be attenuated via inhibition of Hcrt signaling (208), suggesting that the ghrelin-release driven food-seeking behaviors are mediated via the Hcrt system. Taken together, this evidence suggests that dietary hormone release affects the Hcrt system, and that dietary hormone modulation of feeding behaviors is mediated via the Hcrt system. It is clear that motivation for seeking out food sources should be modulated based on the current nutritional status of the animal, and these inhibitory effects of insulin and leptin, paired with the stimulatory effects of ghrelin on the Hcrt system appear to be informing the Hcrt system as to whether or not the animal requires motivation for food-seeking or food-intake behaviors.

### Driving drug seeking behaviors

Given the role Hcrt plays in arousal and motivated states, it is unsurprising that manipulation of Hcrt activity alters many aspects of drug seeking and reward. DiLeone et al. (209) first suggested that Hcrt could be involved in drug abuse, noting that the anatomical projections demonstrated in Peyron et al. (3) implied that these peptides “may also be important in addiction.” Subsequent studies determined that this was indeed the case. For example, chemical activation of LHA Hcrt neurons causes a reinstatement of an extinguished drug-seeking behavior and direct administration of Hcrt to the VTA (a primary reward center in the brain) recapitulates this phenotype (210). It is important to note that Hcrt itself does not drive drug intake intrinsically, but rather drives the motivation to perform behaviors that are associated with drug seeking. Indeed, stress-induced reinstatement of cocaine-seeking behavior is potentiated by Hcrt-1 infusions into the lateral ventricles, an effect dependent on noradrenergic and corticotropin-releasing factor systems (211). Additionally, HcrtR1 antagonism blocks stress-induced reinstatement of previously extinguished drug-seeking behavior, suggesting that the observed effects are due to Hcrt (and not other co-released neurotransmitters) signaling. Supporting this, recent experiments in Hcrt deficient mice demonstrate that they have decreased motivation to perform behaviors required to obtain cocaine rewards (212). Hcrt signaling on VTA DA neurons potentiates neurotransmission via PLC/PKC-dependent insertion of NMDA receptors in VTA DA synapses (213). Additionally, Borgland and colleagues demonstrated that inhibition of HcrtR1 signaling blocks locomotor sensitization to cocaine and attenuates cocaine-induced excitatory currents in VTA DA neurons.

These initial studies have led to a blossoming of research on Hcrt in the context of drug-abuse and addiction, and particularly in relation to the VTA DA system [for review, see Baimel et al. (214)]. Using microdialysis and *in vivo* voltammetry to directly measure dopamine release, España and colleagues demonstrated that Hcrt-1 increases the effects of cocaine on tonic and phasic dopamine signaling, leading to enhanced motivation to self-administer cocaine (215). Others have shown that HcrtR1 blockade reduces work for cocaine self-administration, and Hcrt-1 strengthens presynaptic glutamatergic inputs to the VTA only in rats that have self-administered cocaine or a high fat diet [but not other highly salient cues like aversive stimuli; (180)]. These data suggest that Hcrt selectively potentiates the motivational drive for stimuli providing positive reinforcement (i.e., cocaine, highly palatable diet).

Using an ICSS paradigm in rats, Hollander and colleagues were able to demonstrate that blockade of HcrtR1 decreased nicotine-self administration and motivation to obtain the drug (but not food). Furthermore, they showed that Hcrt fibers innervate the insular cortex, and local blockade of Hcrt signaling in this brain region, but not the adjacent somatosensory cortex, decreased nicotine self-administration (216). This effect seems to be selective to nicotine (or may be species dependent), as Riday and colleagues were unable to demonstrate that HcrtR1 blockade attenuated cocaine self-administration in Swiss-Webster mice (217). More recently, focus has turned to neurotransmitters co-released by Hcrt neurons, including the kappa-opioid receptor (KOR) agonist dynorphin. Muschamp and colleagues demonstrated that Hcrt and dynorphin have opposite effects on the excitability of VTA DA neurons, are contained within the same pre-synaptic vesicles, and Hcrt co-released in the VTA attenuates the dysphoric effects of dynorphin (218). Alongside this evidence, there has additionally been anecdotal evidence that patients with narcolepsy show very low rates of substance abuse, this has recently been observed in Hcrt deficient mice in an experimental paradigm which showed that Hcrt knock out mice have decreased motivation to obtain cocaine as a reward (212). Taken together, these studies suggest a role for Hcrt in modulating drug-seeking behaviors, as well as drug-rewards, there is also a possibility that these neurons are involved in driving motivation for reward-seeking in general, and not just drug-seeking in particular.

### Sexual behavior

While studies investigating the function of Hcrt have focused in on a variety of types of arousal such as arousal from sleeping, hyper-arousal in stress-states, arousal from illicit drug-use, etc. there is also a role for Hcrt in behaviors associated with sexual arousal. While the lateral hypothalamus in general has also been associated with inducing [via electrical stimulation of the LHA, (219)] or impairing [via lesioning of the LH, (220)] male sexual behavior, there is some evidence that LHA Hcrt neurons may be modulating these sex-behaviors. Studies looking at neural activation of Hcrt neurons via Fos expression showed increased Fos-expressing Hcrt neurons after mating (221, 222), and more generally Hcrt neurons also appeared to respond following exposure to either receptive or non-receptive females (222) as well as contextual cues that they have previously learned to associate with sex via conditioned place preference training (223).

Sexual behavior itself can also be modulated via manipulation of Hcrt neurons specifically, by injecting Hcrt-1 into the medial preoptic area significantly improved male sexual behavior by increasing the speed with which males mount females and ejaculate, as well as increasing the frequency with which they mount and ejaculate (224). Conversely, administration of a HcrtR1 antagonist has been shown to impair male sexual behavior across many standard measures such as increasing the mounting and ejaculation latencies, and reducing the frequency of ejaculation, however mounting frequency did not appear to be affected (221). In contrast to the antagonism of HcrtR1, research looking into the targeted lesioning of Hcrt-2 neurons (via Hcrt-2 saporin) has produced mixed results, rats with Hcrt-2 neuron lesions did not appear to show any consistent impairments to the usual measures of male sexual behavior (222, 223). Additionally, the existence of a line of a Hcrt knockout mice (37) would suggest that Hcrts are not required for successful procreation, but may instead be involved in the motivation or initiation of sexual behaviors.

## Integration of motivation and arousal by Hcrt

Hcrt has been shown to play important roles in a wide variety of complex behaviors. The LHA Hcrt system integrates sensory information on stress, metabolic, immune, and motivational states to adjust behavioral responses accordingly. Motivation of behaviors is a two-part process, first comes the energizing component which gives the animal the drive necessary to complete a task, followed by a directional component which determines the type of behavior this burst of energy should be focused toward. The earliest electrophysiological studies in which researchers stimulated neural activity in the LHA showed that activity in this brain region could result in numerous different rewarding behaviors (225–227), which turned out to be more dependent on previous experiences in the same setting (228, 229), rather than topographical placement of the electrodes (230). Considering these findings in the scope of the two sub-processes of motivation, it could be that Hcrt neurons in the LHA are important for the energizing or driving of motivation, and the downstream targets activated by the Hcrt neurons play the role of directing the energy toward context-specific behaviors. Considering that arousal is synonymous with energizing, it could be said that the role of Hcrt in motivated behavior is simply another facet of its numerous functions under the umbrella of arousal. However, further research is required to better understand how the Hcrt circuit modulates different facets of arousal states.

## Conclusion

Research on the Hcrt peptides has been unusually productive. Within just 15 years of their discovery, they were causally implicated in the etiology of narcolepsy, specific receptor antagonists were developed, and a dual-receptor antagonist formulation was FDA-approved for the treatment of insomnia. Using cutting edge tools like CRISPR/Cas9 in tandem with cre-dependent adeno-associated viral vectors to manipulate specific genes within Hcrt neurons or their projection sites will undoubtedly provide further classification of their actions and potential for modulation. While a lot of previous work has focused on the role of Hcrt in mediating arousal specifically, the actual operations and computations of the Hcrt network are still unknown. These computations could be achieved by studying projection-selective subsets of Hcrt neurons. It is still unknown whether individual cells perform the same computations, or whether projection specific subsets perform different computations. In order to develop a better understanding of the intricacies of the Hcrt network it will be important for researchers to investigate this neural circuit with all of these systems in mind. While this can appear complicated—it is very likely that the motivated behaviors and the environmental inputs that drive them are all interconnected and can modulate other relevant behaviors to the Hcrt system. Up until this point the heterogeneity of Hcrt neurons specifically, and lateral hypothalamic neurons in general, have slowed the progress of research in this region. With recent developments in research technologies such as optogenetic stimulation, calcium recordings, single cell RNA sequencing, chemogenetic neuron manipulation, and advances in computational modeling, a better understanding of the Hcrt system is within reach. These methods hold much promise for the next 20 years of hypocretin research to continue to be as fruitful as the first twenty.

## Author contributions

ST and JB wrote the first draft of this manuscript. LdL contributed editorial help and guidance.

### Conflict of interest statement

The authors declare that the research was conducted in the absence of any commercial or financial relationships that could be construed as a potential conflict of interest.
